# Growth Differentiation Factor-15 Deficiency Augments Inflammatory Response and Exacerbates Septic Heart and Renal Injury Induced by Lipopolysaccharide

**DOI:** 10.1038/s41598-017-00902-5

**Published:** 2017-04-21

**Authors:** Palida Abulizi, Neruja Loganathan, Duo Zhao, Tina Mele, Yixin Zhang, Terry Zwiep, Kexiang Liu, Xiufen Zheng

**Affiliations:** 1 Department of Pathology, Western University, London, ON Canada; 2 Department of Cardiology, Xinjiang Medical University, Urumqi, Xinjiang 830000 China; 3 Department of Cardiovascular Surgery, Jilin University, Changchun, Jilin 130000 China; 4 Department of Surgery, Western University, London, ON Canada; 5 London Health Sciences Centre, London, ON Canada; 6 Department of Oncology, Western University, London, ON Canada; 7 Lawson Institute of Health Research, London, ON Canada

## Abstract

Septic acute kidney injury (AKI) and myocardial dysfunction are leading causes of mortality with no accepted method of therapy. In this study we demonstrate the role of growth differentiating factor 15 (GDF15) in septic AKI and myocardial dysfunction using a murine lipopolysaccharide (LPS)-induced sepsis model and an *in vitro* cell culture system. Data show that GDF15 deficiency augments inflammatory response and exacerbates renal and cardiac injury induced by LPS, while over-expression of GDF15 protects the kidney and heart from LPS-induced organ dysfunction. Therefore, this study highlights the therapeutic potential of GDF15 in the treatment of endotoxin-induced sepsis.

## Introduction

Sepsis is a systemic inflammatory response to infection, which causes multiple organ dysfunction including the kidney and heart. Septic acute kidney injury (AKI) and myocardial dysfunction are well- defined complications of severe sepsis and septic shock, remaining as major causes of morbidity and mortality especially in critically ill patients^1, 2^. Even those who recover from the septic shock may present with permanent organ damage^3^. Despite numerous pharmacologic and non-pharmacologic therapies that have been applied clinically for sepsis, there are currently no effective treatments for septic complications in clinical use. Thus, there is a need to identify novel therapeutic targets for the disease.

Although there seems to be a variety of mechanisms involved in the pathogenesis of septic cardiomyopathy and AKI, including ischemia, inflammation, apoptosis and adaptive responses^4^, there is a consensus that an initial inflammatory response is the trigger leading to the decreased filtration function in the kidney^5^. In the kidney and the heart, endotoxins such as LPS, bind to toll-like receptor 4 leading to the activation of the NFκB pathway and the production of cytokines such as TNF-a and IL-17^5, 6^. Inhibitors of inflammatory cytokines, such as anti-TNF-α therapy, seem to be an obvious route to therapy; however, these have failed in clinical trials^7^ and this initial inflammation, although harmful, may be necessary to clear the bacterial insult^5^. Thus, there is a need for therapies that modulate the inflammatory response, instead of those that ablate it completely.

Growth differentiation factor 15 (GDF15), also known as macrophage inhibitory cytokine -1 (MIC-1) and non-steroidal anti-inflammatory drug activated gene-1 (NAG-1), is a divergent member of the transforming growth factor beta family, associated with immunosuppression, anti-apoptosis and anti-inflammation, growth inhibition and cancer cell invasion^8^. It is made as a propeptide that is cleaved in the endoplasmic reticulum and secreted as an active, dimeric protein^9^. In the physiological state, it is known to have an immunosuppressive function in the placenta, where it is thought to reduce fetal exposure to maternal cytokines^10, 11^. GDF15 is upregulated in many disease processes, yet it is unclear whether it leads to the further progression of the disease or provides protection against the disease^11^.

A previous study showed that GDF15 was rapidly up-regulated in response to CCl_4_-induced renal injury, suggesting it may play a role in toxin-mediated kidney injury^12^. A more recent study conducted in LPS-stimulated hNAG-1 transgenic mice showed that decreased levels of pro-inflammatory cytokines were present in the serum, suggesting NAG-1 may play an anti-inflammatory role in response to LPS stimulation via leptin^13^. Contrary to their *in vivo* studies and previous research^9^, *in vitro* studies failed to show that hNAG-1 was directly inhibiting cytokine production in macrophages in response to LPS stimulation^13^. Nonetheless, the impact of GDF15 on septic AKI and cardiomyopathy is unclear.

By using GDF15 KO and transgenic mice and determining their response to LPS-mediated injury, we attempt to elucidate the role of GDF15 in septic AKI and myocardial dysfunction in mice in order to assess its therapeutic potential in preventing sepsis- associated morbidity and mortality. We hypothesized that GDF15 plays a protective role in LPS-induced AKI and cardiomyopathy. In this study, we show the protective role of GDF15 in septic AKI and cardiomyopathy and attempt to determine the potential mechanisms by which GDF15 is eliciting its role, using a murine model of LPS-induced sepsis.

## Results

### GDF15 deficiency exacerbates renal and cardiac dysfunction induced by LPS stimulation

To investigate the impact of GDF15 on septic AKI and cardiomyopathy, we treated mice intraperitoneally with 4 mg/kg LPS in PBS or PBS only as a control to induce septic response; this is a commonly used animal model for the study of sepsis^14^. Sixteen hours later, we measured renal function and heart function.

**Figure 1:**
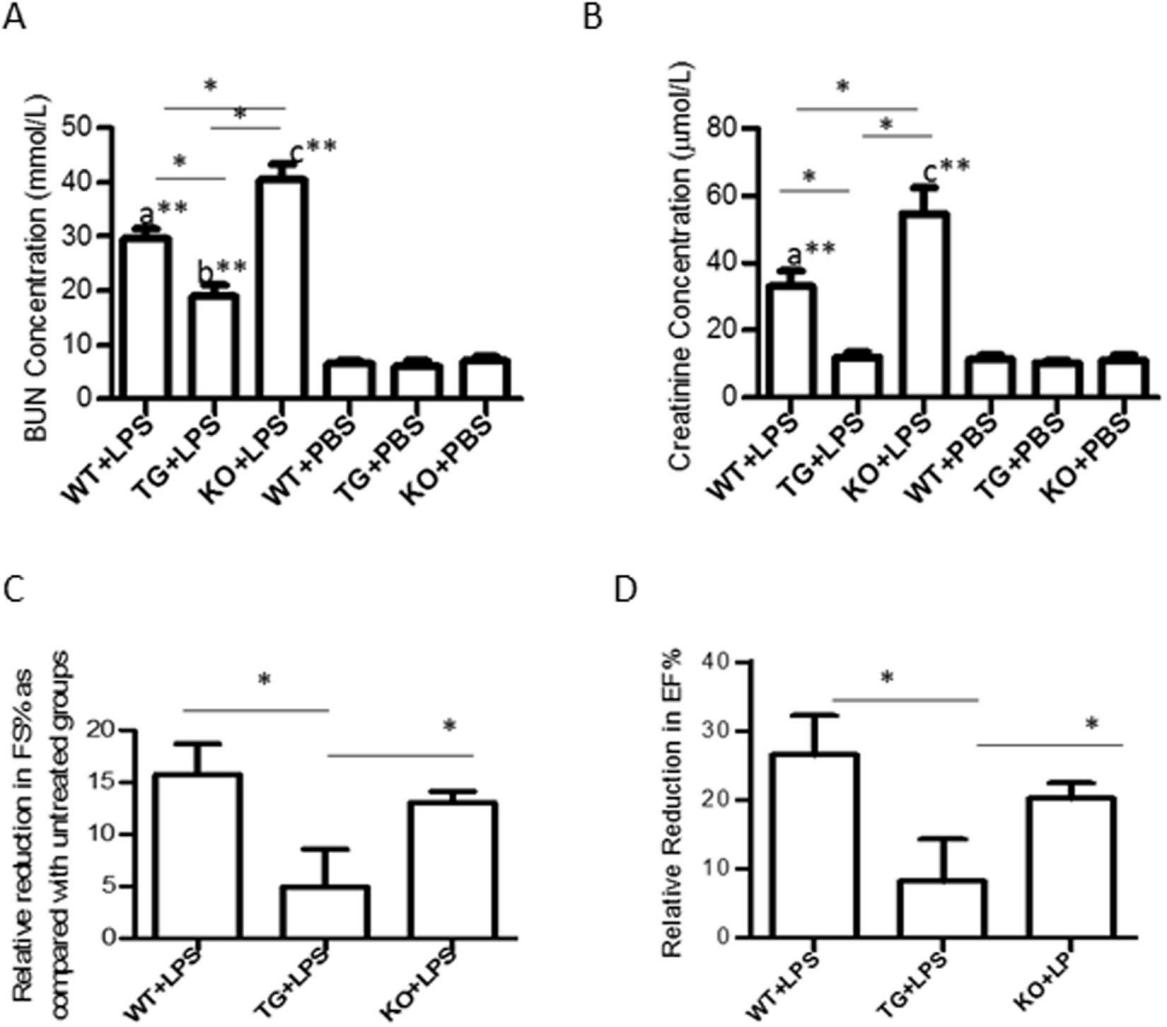
GDF15 deficiency exacerbates cardiac and renal injury after LPS administration. WT, GDF15 TG and GDF15 KO mice were i.p. injected with 4 mg/kg of LPS (WT + LPS, TG + LPS and KO + LPS group) or PBS (WT, TG and KO group) for 16 hours. Blood was collected to measure concentrations of BUN (**A**) and serum creatinine (**B**) to assess renal function Ultrasound scanning was performed to measure EF% (**C**) and FS% (**D**) for heart function. After ultrasound scanning, mice were sacrificed. n = 9–10 for mice treated with LPS and n = 4/group for PBS injected groups in Fig. 1(A,B); n = 5 for (**C**,**D**); *P ≤ 0.05, **P ≤ 0.01. a: WT + LPS vs. either WT, TG or KO group; b: TG + LPS vs. either WT, TG or KO group; c: KO + LPS vs. either WT, TG or KO group.

Renal function was assessed by measuring levels of blood urea nitrogen (BUN) and serum creatinine since AKI is characterized by a decrease in the filtration function of the kidney and is detected using serum creatinine levels and BUN levels^3^. Figure 1A,B illustrates the levels of BUN and creatinine across all treatment groups. Increased levels of these markers indicate reduced renal function. We found that BUN levels were significantly (p ≤ 0.01) increased in the LPS treated groups compared to the control (PBS) groups, suggesting LPS stimulation induced renal function decline (Fig. 1A). The serum creatinine levels in the LPS injected WT and KO groups were significantly increased compared with their PBS injected control groups, whereas the creatinine levels did not significantly increase in the LPS injected TG group as compared with the PBS injected control groups (Fig. 1B). Furthermore, we found that mice expressing different levels of GDF15 displayed different degrees of renal injury in response to the LPS insult. GDF15 KO mice had significantly (p ≤ 0.01) greater levels of both BUN and creatinine compared to WT mice (Fig. 1A,B). In contrast, GDF15 TG mice had significantly lower levels of BUN compared to WT and GDF15 KO mice after LPS stimulation. A similar reduction was seen in the creatinine levels of GDF15 TG mice. Consequently, deficiency of GDF15 exacerbated renal injury caused by LPS-insults, while overexpression of GDF15 prevented the renal function decline.

Echocardiography was performed to detect heart function of mice. LV fractional shortening (FS) and ejection fraction (EF) were used as indices of cardiac contractile function. There are no significant differences at the basal EF% and FS% among PBS injected WT, KO or TG mice with EF% (>50%) and FS% (25–50%), despite KO mice experiencing a relatively low EF% and FS% (data not shown). Treatment with LPS significantly reduced FS% and EF% in comparison with PBS only treated groups, confirming LPS *i.p*. injection caused heart function declination. As GDF15 KO mice had a slightly lower basal EF% and FS% compared to that of WT or TG mice, we used reductions of EF% and FS% (EF% _PBS-treated_- EF% _LPS-treated_, and FS% _PBS-treated_- Fs% _LPS-treated_) to assess the effect of GDF15 on LPS-induced changes to heart function. As shown in Fig. 1C, the reduction of EF% in the TG mice was significantly less than that of WT mice or KO mice, indicating that over expression of GDF15 can protect heart function from endotoxin- induced injury. There was also a smaller reduction in FS% in TG mice, but it did not reach statistical significance (Fig. 1D). There were no profound differences in the reduction of EF% and FS% between WT mice and GDF15 KO mice.

### GDF15 deficiency induces more severe LPS-mediated injury in the kidneys and heart

**Figure 2:**
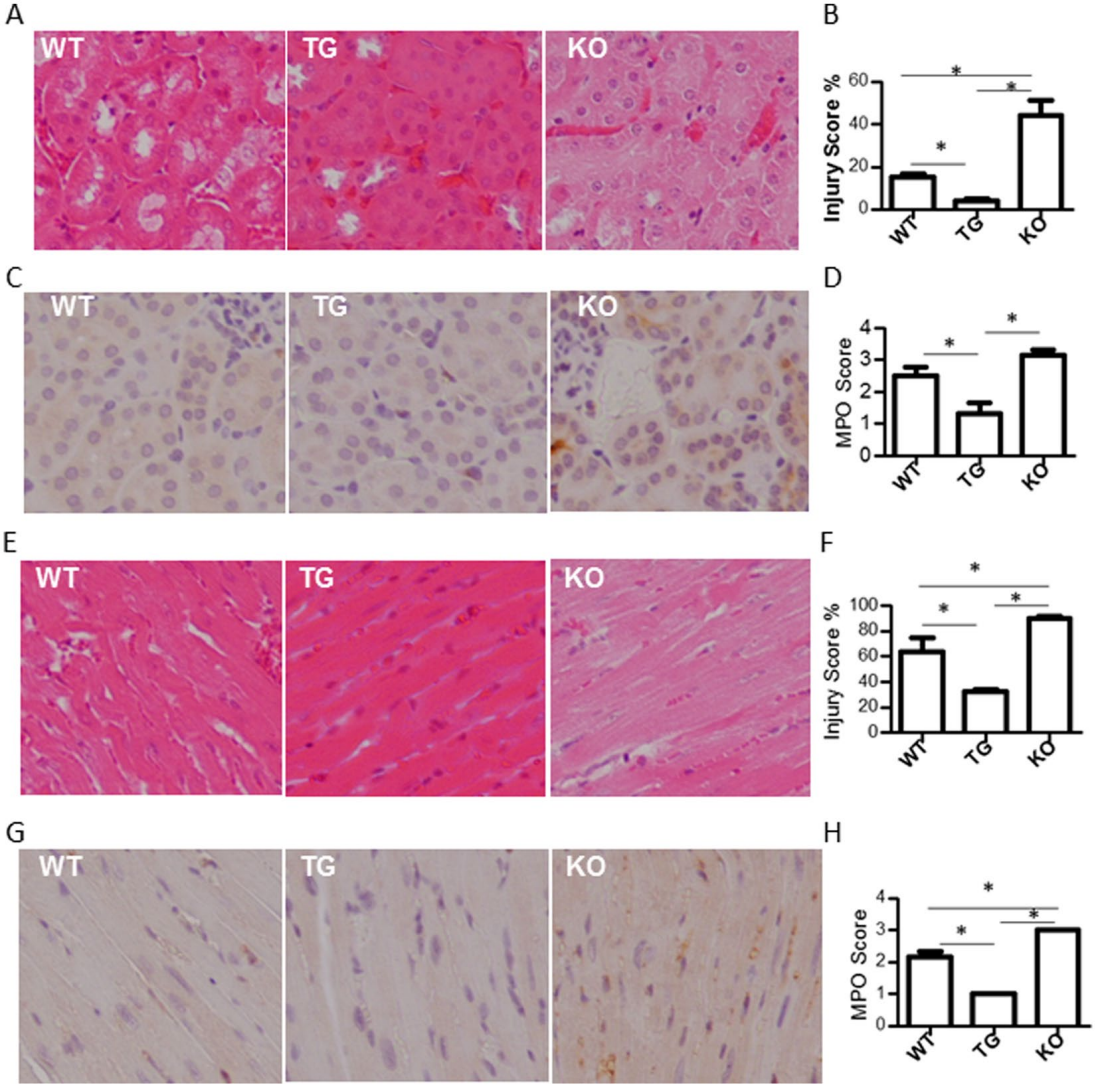
GDF15 KO mice show greater levels of tubular damage and cardiac injury, and increased neutrophil infiltration. Sixteen hours after LPS treatment, kidneys and hearts were harvested from mice. Tissues from all groups were sectioned and stained with H&E and detected MPO activity. Images were taken from kidney cortex at 200 X magnification. (**A**) Representative HE staining images of kidney tissues. (**B**) Injury score for kidney tissues. (**C**) Representative MPO images of kidney tissues. (**D**) MPO score for kidney tissues. (**E**) Representative HE staining images of heart tissues. (**F**) Injury score for heart tissues. (**G**) Representative MPO images of heart tissues. (**H**) MPO score for heart tissues. n = 6 for HE staining. n = 4 for MPO assays. *P ≤ 0.05.

To further demonstrate the effect of GDF15 in LPS-induced renal and cardiac injury, kidney and heart tissues were harvested from the LPS-treated mice and subjected to histopathological examination. Figure 2A shows the representative H&E-stained sections of the kidneys and Fig. 2B shows the severity of the injury. We found that pathological changes occurred in the proximal tubules as these are known to be most affected during sepsis. Changes in the kidneys were hydropic degeneration and necrosis. We observed diffuse swelling (hydropic degeneration) across all treatment groups, whereas there were significant differences in necrosis between the groups. Kidneys from LPS-treated TG mice had visible brush borders indicating decreased severity of damage. In contrast, the kidneys from LPS injected KO mice had a greater degree of necrosis compared to other groups (Fig. 2A,B). We also detected neutrophil infiltration in the kidney using the MPO assay. We found that more neutrophils infiltrated in kidneys from GDF15 KO mice than either WT or GDF15 TG mice (p ≤ 0.05) (Fig. 2C,D). A reduction of MPO positive cells was seen in GDF15 TG mice (Fig. 2C,D). This suggests that the absence of GDF15 leads to a higher injury severity.

We also observed that LPS caused pathological changes including hydropic degeneration, gradual degeneration and necrosis in the myocardium (Fig. 2E,F). Hearts from LPS injected WT mice showed that more than 60% of myocardium were necrotic and moreover, over 90% were necrotic in LPS-injected KO mice. In contrast, only about 30% myocardium were necrotic in GDF15 TG mice. These data indicate that deficiency of GDF15 promotes LPS-induced cardiomyocyte injury, while over expression of GDF15 reduces injury.

LPS injection also induces neutrophil infiltration in the heart^15^. We detected MPO activities in heart tissues treated with LPS. As shown in Fig. 2G,H, MPO activity was clearly observed in heart tissue sections from WT and KO mice, indicating neutrophil infiltration. Knockout of GDF15 increased neutrophil infiltration whereas overexpression of GDF15 reduced infiltration of neutrophils in the hearts.

### GDF15 deficiency leads to increased apoptosis in kidney and heart tissues after LPS administration

**Figure 3:**
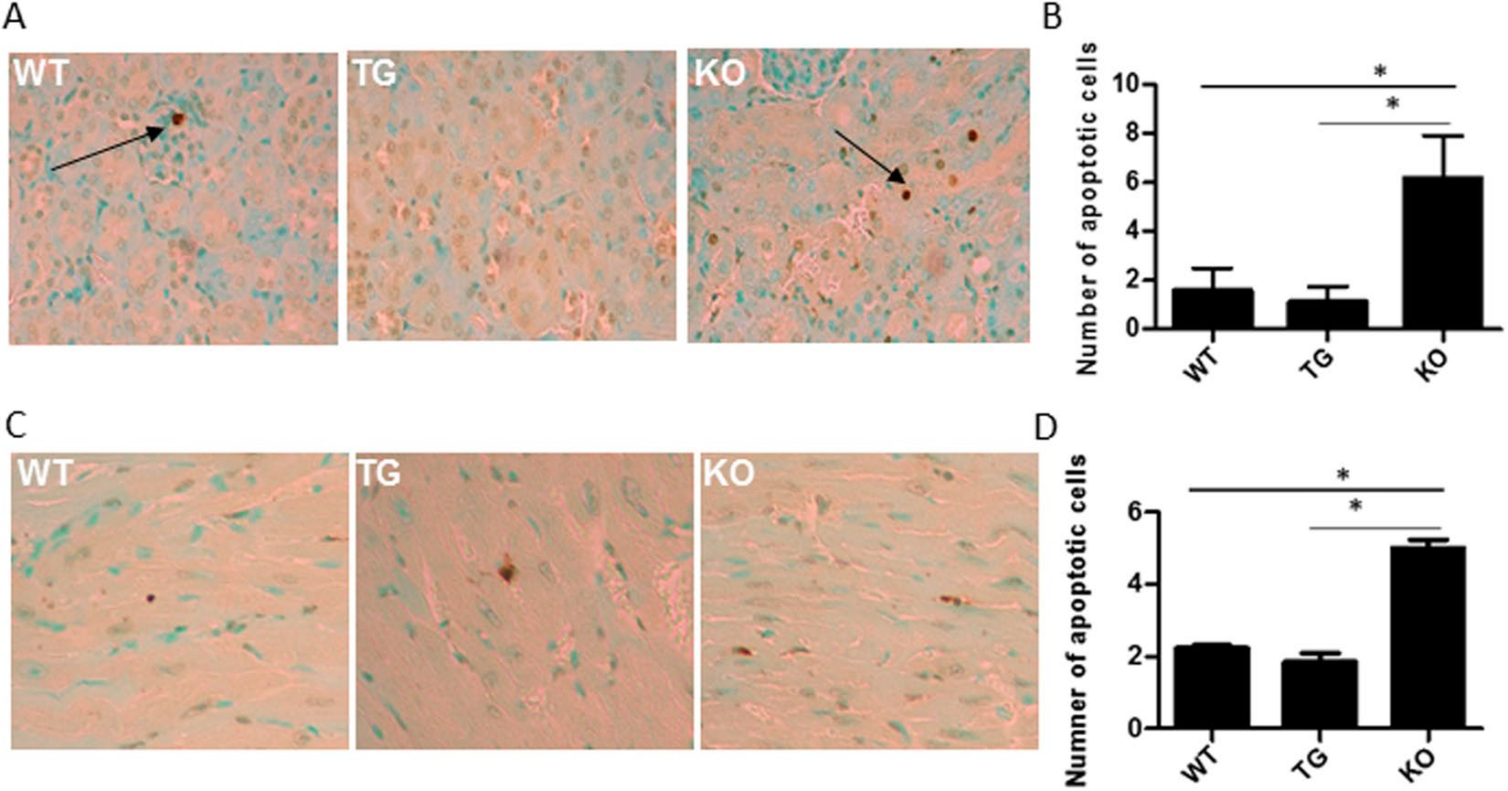
GDF15 KO mice have increased levels of apoptotic cells in kidney (**A,B**) and heart tissues (**C,D**). Formalin fixed, paraffin embedded sections of GDF15 WT, TG and KO mice treated with LPS were stained for the TUNEL assay. The number of stained cells were counted for each slide across ten sections at 200 X magnification and averaged. (**A,C**) are representative images. (**B,D**) are summarized data. n = 4;*P ≤ 0.05, **P ≤ 0.01, ***P ≤ 0.001.

Apoptosis is one of mechanisms by which LPS can lead to kidney and heart cell injury [3–5, 15]. Accordingly, we determined the levels of kidney and heart cell apoptosis in the kidney and heart tissues of LPS treated mice using the TUNEL assay. Representative kidney sections, viewed at 200 X magnification are shown in Fig. 3A. TUNEL positive cells were primarily seen in the proximal tubules and to a lesser degree, in the glomerulus (Fig. 3A). GDF15 KO mice showed significantly greater numbers of TUNEL positive cells (p ≤ 0.01) compared to WT and GDF15 TG mice (Fig. 3B). There was a reduction in apoptotic cell numbers in TG mice in comparison with WT, but the difference was not statistically significant.

Representative heart sections, viewed at 200 X magnification are shown in Fig. 3C. TUNEL positive cells were seen in the heart tissue (Fig. 3C). There were more apoptotic cells seen in GDF15 KO mice (p ≤ 0.01) compared to WT and GDF15 TG mice (Fig. 3D). Similar to kidney tissue, there were fewer apoptotic cells in TG mice compared with WT, but the difference was not statistically significant.

## GDF15 deficiency increases mortality induced by endotoxin

**Figure 4:**
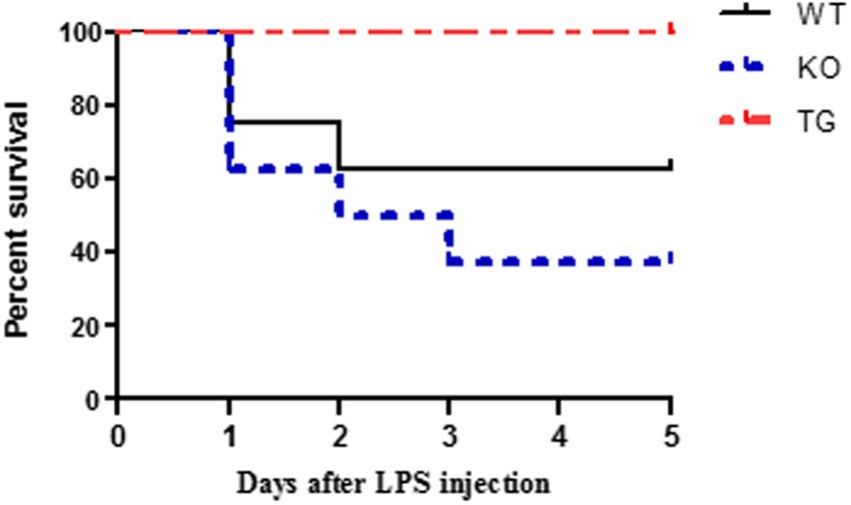
Over expression of GDF15 prevents LPS-induced mortality of animals. WT, TG and KO mice (n = 8) were *i.p*. injected with 20 mg/kg LPS. Animal health and survival was observed every 12 h in for 5 days after LPS injection.

Over exposure of endotoxin LPS is known to cause animal death. Eight mice per group were *i.p*. injected with 20 mg/kg LPS. Animal survival was observed for five days after LPS injection. As shown in Fig. 4, three mice in the WT group died within the first three days, and 5 mice died in the KO group. All mice in both WT and KO group appeared very sick with scruffy hairs, dehydration and decreased food intake, and decreased movement. These symptoms lasted for 4 days. In contrast, all mice in the TG group survived. Mice in the TG group looked weak only on the first day after LPS injection and recovered to a normal level of activity by day 2. There was a significant difference in animal survival among the groups (*P* = 0.048) compared by the Log-rank (Mantel-Cox) Test.

### Lack of GDF15 upregulates the expression of inflammatory cytokines induced by LPS stimulation

**Figure 5:**
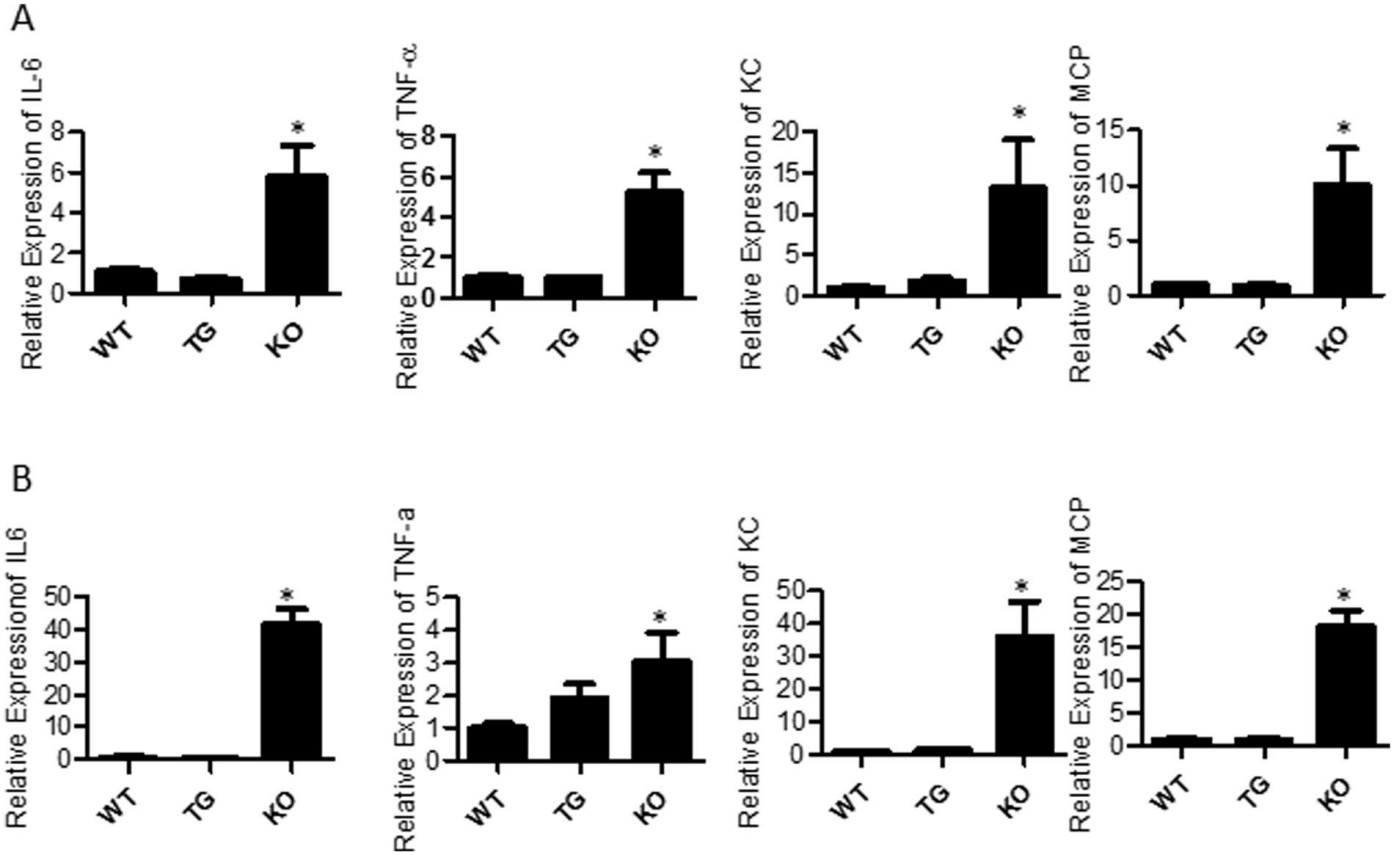
Inflammatory gene expression in kidney and heart tissue. WT, TG and KO mice were *i.p* injected with LPS. RNA was extracted from the cortex of the kidney and heart 16 h after LPS treatment. qRT-PCR was performed using SYBR green to detect the expression of IL-6, TNF-α, KC and MCP-1 and the housekeeping gene GAPDH was used as the internal control. Data is expressed using the ∆∆CT method and normalized to WT + LPS. (**A**) Kidney tissues (**B**) Heart tissues. n = 3–5 = ;*P ≤ 0.05.

LPS stimulation induced expression of inflammatory genes [3–5, 15]. Gene expression of inflammatory cytokines in the kidney cortex of mice treated with LPS or PBS were determined using qRT- PCR. Figure 5A,B show the relative expression of various genes across the different treatment groups, the WT mice treated with LPS were used as the normalizer. We found that the expression of inflammatory mediators or cytokines MCP-1, KC, IL-6 and TNF-α was up-regulated in the kidney and heart tissues from the three strains of mice (Fig. 5A,B). Compared to WT or GDF15 TG mice, GDF15 KO mice treated with LPS expressed significantly higher levels of MCP-1, KC, IL-6 and TNF-α (Fig. 5A,B).

### Pre- treatment with rhGDF15 significantly reduces cell apoptosis induced by LPS *in vitro*

**Figure 6:**
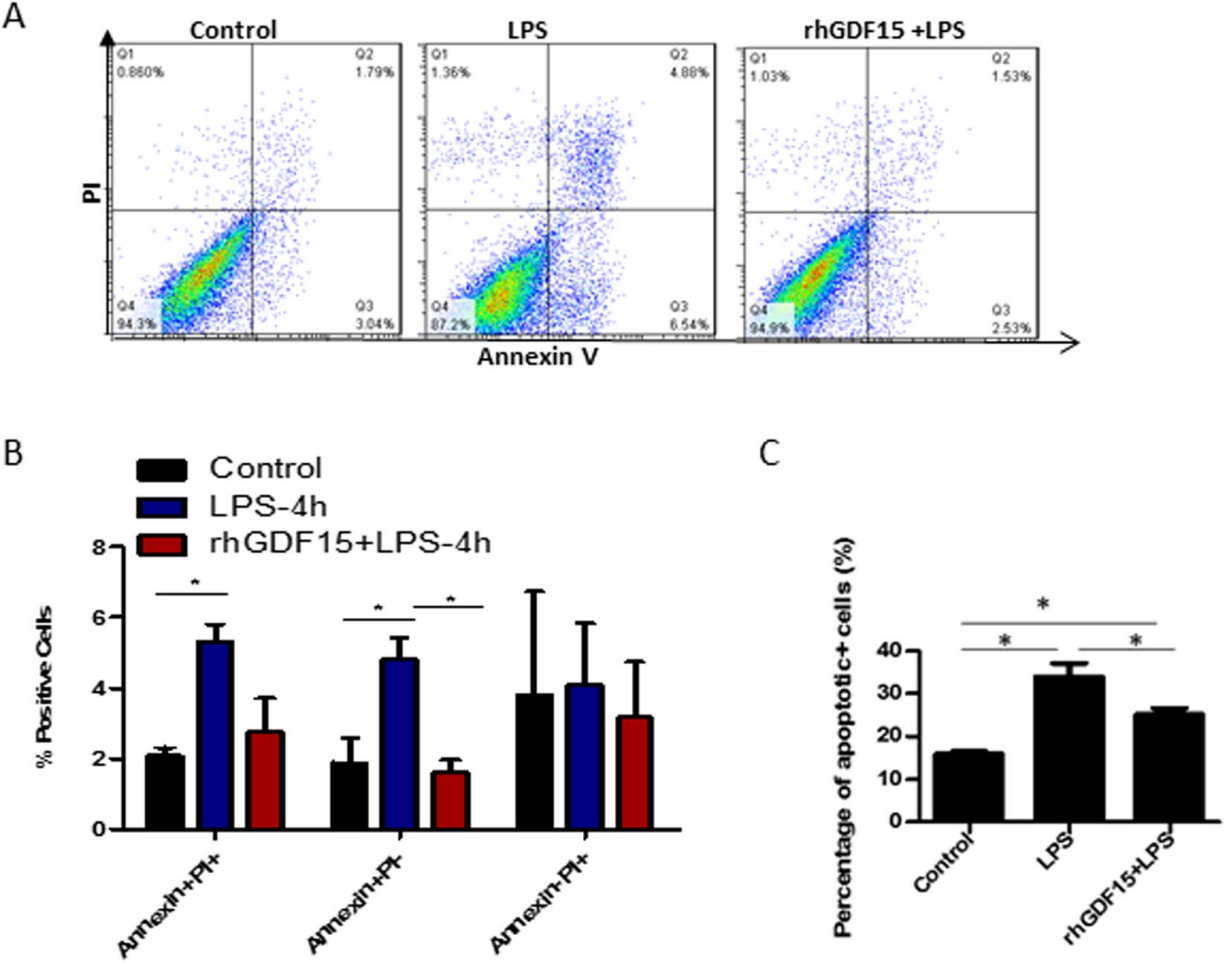
Prior treatment with rhGDF15 reduces the amount of early apoptotic cells seen 4 hours after LPS treatment. Primary kidney tubular cells and primary cardiomyocytes from WT mice were cultured and treated with 25 µg/ml LPS alone for 4 hs, rhGDF15 two hours prior to LPS treatment or were untreated. Cells were then collected, stained with Annexin V and PI and detected using flow cytometry. Representative dot plots are shown for each treatment group (**A**). Summarized data for kidney cells are shown in (**B**) and for heart cells in (**C**). Data are representative of 3 independent experiments; *P ≤ 0.05.

To further confirm the protective effects of GDF15 on LPS-mediated kidney and cardiac injury, we conducted *in vitro* experiments using rhGDF15 to treat primary kidney tubular cells and heart cells. We cultured primary kidney tubular cells from WT mice and treated them with rhGDF15 2 hours prior to a 4 h LPS stimulation. Cells were then collected and stained with Annexin V and PI and analyzed using flow cytometry. As shown in Fig. 6A,B, LPS treatment significantly increases the percent of double positive (p ≤ 0.05) and Annexin V + (p ≤ 0.05) cells, suggesting LPS treatment leads to increased levels of tubular cell apoptosis. Pre-treatment with rhGDF15 significantly reduced (p ≤ 0.05) the levels of Annexin V + cells, but not double positive cells, although a decreasing trend was seen.

To investigate the impact of exogenous rhGDF15 on cardiomyocytes, we isolated and cultured primary cardiomyocytes from neonatal WT mice and treated these cells with rhGDF15 before adding LPS. Cell apoptosis was detected by double staining with Annexin V and PI and followed by flow cytometry analysis. As shown in Fig. 6C, LPS treatment significantly increased the percent of apoptotic (p ≤ cells (p ≤ 0.05), suggesting LPS treatment leads to increased levels of cardiomyocyte apoptosis. Pre-treatment with rhGDF15 significantly reduced (p ≤ 0.05) the levels of apoptotic cells.

## Discussion

This study, for the first time, demonstrates the impact of GDF15 on AKI and myocardial dysfunction induced by LPS and its potential therapeutic application in attenuating renal and cardiac injury. We have also demonstrated that GDF15 plays a protective role in LPS-induced AKI and myocardial dysfunction by reducing apoptosis and the inflammatory response.

Septic AKI and cardiomyopathy are leading causes of mortality with no currently well-accepted methods of therapy^4^. Endotoxins such as LPS from bacteria, interact through toll-like receptor 4 on immune cells, heart cells, and proximal tubular cells, activating the NFκB pathway and the production of cytokines^4, 6^. These cytokines induce the production of nitric oxide, which plays a role in directly destroying the tubular epithelial and cardiac cells^16^. Furthermore, it is suggested that initially the cytokines lead to an inflammatory response characterized by the cytokine storm, adaptive immunity involvement and organ infiltration of immune cells^4^. The tubular cells and myocytes then adopt an immunosuppressed state of decreased lymphocyte proliferation and eventually undergo apoptosis^4^, leading to the decreased filtration function seen in AKI and heart failure. Consequently, there is a need to discover molecules that modulate the inflammatory response seen in septic organ failure.

GDF15 has pleiotropic functions in both the physiological and pathological conditions^11^. For instance, the high expression of GDF15 in the placenta during pregnancy protects the fetus from the activity of maternal cytokines and prevents miscarriage, illustrating its immunosuppression^11, 17^. Similarly, elevated levels of GDF15 are associated with reduced disease severity in chronic inflammatory diseases^18^, including rheumatoid arthritis^18, 19^ and atherosclerosis^18^, suggesting an anti-inflammatory role for GDF15, although there are controversial results about anti-atherosclerosis. GDF15 has been reported as an immediate response gene in response to stress and its over expression aims to prevent further damage^20–25^. The anti-apoptotic function of GDF15 has been demonstrated in cardiac ischemia-reperfusion injury models^21, 26^. Results show that treatment of oral squamous cell carcinoma cell lines with GDF15 significantly reduced the activity of caspase 3/7^27^ which also demonstrated an anti-apoptotic role of GDF15, despite that GDF15 is known to have anti-tumorigenic activity due to its pro-apoptotic activity^18, 28^. Our study suggests that GDF15 is involved in septic AKI and cardiomyopathy and protects the kidney and heart from LPS-induced injury, potentially by acting as an anti-apoptotic and anti-inflammatory mediator.

LPS-mediated AKI is generally characterized by apoptosis of proximal tubular epithelial cells and neutrophil infiltration^29^. Thus, we investigated the role of GDF15 in these processes after LPS stimulation. GDF15 deficiency led to increased levels of apoptosis and neutrophil infiltration *in vivo*, suggesting that GDF15 may be eliciting its protective role by acting as an anti-inflammatory and anti-apoptotic mediator. In a model of myocardial infarction, Kempf *et al*. showed that administration of recombinant GDF15 protein to mice deficient in GDF15 led to reduced polymorphonuclear leukocyte (PMN) recruitment to the site of injury^24^. GDF15′s ability to prevent excessive neutrophil recruitment was attributed to its ability to block activation of β2 integrin and subsequently PMN adhesion to ICAM-1^24^. In this study, we found there was reduced neutrophil infiltration in heart tissues from TG mice. These findings suggest that the presence of GDF15 in WT and GDF15 TG mice may have prevented excessive neutrophil recruitment into kidney tissues after LPS stimulation.

IL-6, KC, TNF-α and the chemokine MCP-1 which recruits monocytes to the site of injury in kidney and heart tissues after the LPS insult have been shown to be upregulated after LPS stimulation in previous studies^30, 31^. To further assess the mechanism by which GDF15 is modulating inflammation in sepsis, we evaluated these cytokine expressions. Our study showed significantly greater levels of cytokines in LPS-treated KO mice compared to WT mice. A study looking at the role of TNF- α in LPS-induced AKI showed that TNF-α produced both by immune cells systemically and by tubular cells locally acts via tumour necrosis factor receptor 1 to induce damage through apoptotic and inflammatory pathways following LPS stimulation^29^. Thus, the increased levels of TNF- α, IL-6, KC and MCP-1 in our *in vivo* GDF15 KO mice suggest that the absence of GDF15 induced further damage due to increasing expression of these mediators. These findings support *in vitro* studies in macrophages reported by Bootcov MR *et al*., showing GDF15′s ability to supress TNF- α production^9^. They are also align with the results reported by Kim group that hNAG-1 transgenic mice had decreased levels of pro-inflammatory cytokines in sera after LPS treatment as compared to WT mice^13^.

To further confirm the effects of GDF15 on kidney tubular cells and myocardium, we performed *in vitro* studies using primary kidney tubular cells and cardiomyocytes. Our *in vitro* experiments showed that treatment of cultured primary kidney tubular cells with rhGDF15 prior to LPS administration is able to reduce the levels of MCP-1 and TNF-α, to a certain extent, seen after the LPS insult (data not shown). These findings further suggest that GDF15 protects kidney cells from LPS-mediated injury by modulating the levels of these mediators.

TNF- α and MCP-1 are upregulated in response to LPS-stimulation via the NFκB pathway^6, 31^. In rats, deletion of the NFκB binding sites in the enhancer regions of the MCP1 gene resulted in a loss of LPS-induced gene activity^31^. GDF15 has been shown to block NFκB activity in many cell types, including prostate cancer cells, osteoclasts and colon cancer cells^8, 32^, and it is possible that it performs its role in LPS-mediated AKI and myocardial dysfunction by blocking the NFκB-dependent production of cytokines. It is suggested that NFκB inhibition with GDF15 overexpression may be mediated by prolonged activity of IkB, a molecule that inhibits NFκB subunits^32^. These findings suggest that GDF15 has the ability to block NFκB activity and this may be the mechanism by which GDF15 is acting to supress inflammatory cytokines in LPS-treated primary kidney tubular cells. In our study, we also observed increased activation of NFκB signaling stimulated by LPS *in vitro* and *in vivo*. This level was reduced when cells were pre-treated with rhGDF15 (data not shown). Further experiments are needed to confirm the potential mechanism. Regardless, our study shows that GDF15 has the ability to reduce inflammation after LPS stimulation, specifically, in kidney tubular cells.

Our *in vitro* studies also showed that treatment of both primary kidney tubular cells and primary cardiomyocytes with rhGDF15 prior to LPS stimulation significantly reduced the number of apoptotic kidney and heart cells. Our study indicates that LPS caused apoptosis of kidney and heart cells and GDF15 plays a role in reducing the levels of apoptosis seen after an LPS insult, both *in vivo* (TUNEL) and *in vitro*. rhGDF15 may reduce apoptosis *in vitro* by preventing caspase cleavage (data not shown). These findings agree with previous studies regarding the anti-apoptotic role of GDF15 in protecting cardiomyocytes from apoptosis^11, 21, 26, 33^.

In summary, our study is the first to show that GDF15 plays a protective role in LPS-induced septic renal and cardiac organ damage. Particularly, GDF15 acts to reduce the levels of apoptosis and neutrophil infiltration in kidney and heart tissues after an LPS insult. Furthermore, GDF15 reduces the levels of inflammatory cytokines IFN-γ, IL-6, MCP-1 and TNF-α, stimulated by LPS. Thus, GDF15 may serve its protective function by acting as both an anti-apoptotic and anti-inflammatory mediator. Consequently, GDF15 may have therapeutic potential for the treatment of septic AKI and cardiomyopathy.

## Materials and Methods

### Animals

C57B/6 wild type mice were purchased from Charles River Laboratories (Canada). Whole genome GDF15 knock-out (KO) mice, generated on a C57BL/6 background using standard gene-targeting techniques were kindly provided by Professor Se-Jin Lee at John Hopkins University (Baltimore, MD)^34^. GDF15 Transgenic (TG) mice generated on a C57BL/6 background and ubiquitously expressing high levels of human GDF15 (hNAG1) under the control of the chicken *β*-actin promoter (CAG) were kindly provided by Dr. Eling (*National Institutes of Health*, NIH) and Dr. Baek at the University of Tennessee (Knoxville, TN, USA)^13, 28^. All experiments in the study were performed in accordance with the guidelines established by the Canadian Council of Animal Care and were approved by the Animal Care Committee of the University of Western Ontario.

### Induction of sepsis and collection of tissues

Eight-week old mice received an intraperitoneal injection of 4 mg/kg LPS (Sigma-Aldrich, St. Louis, MO) to induce sepsis. Mice receiving phosphate buffered saline (PBS) injections served as the control. Sixteen hours after LPS injection, mice were sacrificed by CO inhalation and the blood, heart and kidney tissues were collected. Tissues from each mouse were used for histological analysis and were stored at −80 °C for use in gene expression studies.

### Measurement of Renal Function

Renal function was assessed by levels of serum creatinine and BUN. Blood was centrifuged for 20 minutes at 10,000 rpm and the serum was collected. Serum samples were delivered to the London Health Sciences Centre Core Laboratory (London, ON) to measure BUN levels and serum creatinine levels.

## Echocardiography

Mice were lightly anesthetized using continuous delivery of the gas inhalation agent isoflurane. (1) Hair on the chest was shaved. Mice were then placed on a warm pad to maintain body temperature around 37 °C. ECG needle leads were connected to the limbs for electrocardiogram gating. Animals were scanned using a 40 MHz linear array transducer (MS-550D, Visual Sonics, Toronto, Canada) attached to an ultrasound system (Vevo 2100, Visual Sonics) with a nominal in-plane spatial resolution of 40 μm (axial) × 80 μm (lateral). M-mode and 2-D parasternal short-axis scans (133 frames/s) at the level of the papillary muscles were used to measure changes in left-ventricular (LV) end-systolic inner diameter (LVIDs), LV end-diastolic inner diameter (LVIDd), LV posterior wall thickness in end-diastole (LVPW;d), and end-systole (LVPW;s). LV volumes at end-diastole (LVEDV) and end-systole (LVESV) were calculated using the formulas: LVEDV = [7/(2.4 + LVIDd)] × [LVIDd3] and LVESV = [7/(2.4 + LVIDs)] × [LVIDs3]. LV fractional shortening (FS) and ejection fraction (EF) were used as indices of cardiac contractile function and were calculated from the inner diameters according to the formula: FS [%] = (LVIDd-LVIDs)/LVIDd × 100, and from LV volumes according to the formula: EF [%] = (LVEDV-LVESV)/LVEDV × 100, respectively^35^.

### Histological Analysis

Hearts and Kidneys were fixed in 10% formalin and processed for histological examination using standard techniques. Formalin-fixed tissues were embedded in paraffin, cut into 5 µm sections and stained with H&E. Stained sections were examined under a light microscope (Nikon Eclipse 90i Automated Microscope) for tubular damage and representative images of the kidney cortex were taken at 200 X magnification. The criteria to detect tubular damage included the loss of the brush border in the proximal tubule epithelium, hydropic degeneration and necrosis. The criteria to detect cardiomyocyte damage included gradual degeneration and necrosis. Injury score was determined by overall injury based on the area of necrosis.

### Terminal Deoxynucleotidyl Transferase-Mediated dUTP Nick-End Labelling (TUNEL) Assay

Cell apoptosis in the kidneys of LPS-treated mice was detected by the TUNEL assay using paraffin embedded tissue sections and an *in situ* cell death detection kit according to the manufacturer’s instructions (Roche, Mississauga, ON). Haematoxylin was used as the counterstain. In the TUNEL assay, the nucleus of the apoptotic cells was stained brown. Ten non-overlapping fields of each section were visualized at 200 X magnification under a light microscope and the number of apoptotic cells were counted in each field and averaged across each section.

### Myeloperoxidase (MPO) Activity

Neutrophil infiltration in kidney tissues of LPS treated mice was examined by detection of MPO activity. Immunohistochemistry was performed using standard protocols. Paraffin-embedded tissue sections were deparaffinised, rehydrated, blocked and incubated with a polyclonal rabbit MPO antibody (1:100, NeoMarkers, Fremont, CA). This was followed by incubation with EnVision + anti-rabbit-HRP (Dako, Carpinteria, CA). Sections were then incubated with the chromogenic substrate and counterstained with hematoxylin. Each section was visualized at 200 X magnification under a light microscope. MPO activity was semi-scored using a five point system based on area of involvement as follows: 0, <10%; 1, 10% to 25%; 2, 25% to 50%; 3, 50% to 75%; and 4, 75% to 100%.

### RNA Extraction and qRT- PCR

Gene expression analysis was performed using 3–5 mice in the LPS treated groups. Total RNA was extracted from kidney tissues or cultured cells using TRIzol (Invitrogen Life Technologies, Carlsbad, CA) according to the company’s instructions. The first strand complementary DNA was synthesized by incubating 3 μg of total RNA, 0.5 μg oligo(dT) and 200 U/μL of M-MuLV Reverse Transcriptase (New England BioLabs, Inc., Whitby, ON) for 50 minutes at 42 °C in the presence of 10X M-MuLV Reverse Transcriptase buffer and 10 mM dNTP mixture (Life Technologies). Quantitative real time PCR reactions were performed using the CFX Connect Real-Time System (BioRad) in a 10 μL volume with 2X SensiFAST SYBR No-ROX Mix (BioLine, FroggaBio Inc, Toronto, ON). Reactions were performed for 40 cycles using the following temperature profile: 95 °C for 2 min (initial denaturation); 95 °C for 10 s (denaturation); 60 °C for 10 s (annealing); 72 °C for 20 s (extension). Data were analyzed using the ΔΔCT method and reported as relative expression using mouse glyceraldehyde-3-phosphate dehydrogenase (GAPDH) as the housekeeping gene. The following primer sequences were used: MCP-1, 5′-aggtccctgtcatgcttctg-3′(F) and 5′-tctggacccattccttcttg-3′ (R); TNF-α: 5′-cgtcagccgatttgctatct-3′ (F) and 5′-cggactccgcaaagtctaag-3′(R); KC: 5′-gcacccaaaccgaagtcata-3′ (F) and 5′-tggggacaccttttagcatc-3′(R); IL-6: 5′-ccggagaggagacttcacag-3′ (F) and 5′-ggaaattggggtaggaagga-3′(R); GAPDH: 5′-caggagcgagaccccactaacat-3′′ (F) and 5′-gtcagatccacgacggacacatt-3′(R).

### Primary renal tubular cell culture

Kidneys were isolated from new born C57BL/6 mice and diced. Tissue was digested by incubation in 1 mg/ml collagenase type v (Sigma) and K1 media for 30 minutes at 37 °C. The resulting suspension was filtered through a cell strainer (mesh opening 40 µm, BD Falcon, Mississauga, ON) and the filtered suspension was centrifuged at 1600 rpm for 5 minutes. The pellet was suspended in K1 media and transferred to a collagen (10 μg/ml, Sigma) coated 75 cm^2^ culture flask. Cells were passaged upon reaching 70–80% confluence. Cells used for the subsequent experiments were at passage number 3, 4 or 5.

K1 media consisted of DMEM:F12 (1:1 volume, Invitrogen, Carlsbad, CA) supplemented with 25 μg/ml epidermal growth factor (Sigma), 1 M HEPES (Invitrogen), penicillin/streptomycin (Invitrogen), 5% fetal bovine serum (Invitrogen) and a hormone mixture (sodium selenite, insulin, apo-transferrin, hydrocortisone, triiodothyronine (Sigma) in HBSS/HEPES).

### Primary cardiomyocyte isolation

Hearts were isolated from new born C57BL/6 mice and diced. Tissue was digested by incubation in 25 µg/ml Libase (Roech Life Science) and D-Hanks’s buffer for 40 minutes at 37 °C. The resulting suspension was filtered through a cell strainer (mesh opening 40 µm, BD Falcon, Mississauga, ON) and the filtered suspension was centrifuged at 200 × g for 10 minutes. The pellet was suspended in DMEM media and transferred to gelatin (1%, Sigma) coated 12 well plates.

### rhGDF15 treatments and LPS stimulation *in vitro*

Cultured primary cardiomyocytes or kidney tubular cells were plated in 12 well plates pre-coated with 1% gelatin (Sigma) at a density of 1 × 10^5^ cells and grown overnight in K1++ media (for kidney cells). Culture medium was replaced the following morning and wells were separated into three treatment groups: a. 25 ng/ml rhGDF15 (R&D systems Inc, Minneapolis, MN) was added to the media 2 hours prior to adding 25 μg/ml LPS for 4 hours; b. 25 μg/ml LPS for 4 hours; c. no treatment. Cells were collected from the three treatment groups (control, LPS alone, rhGDF15 + LPS) to detect apoptosis and for RNA extraction.

### Cell Apoptosis

Cultured primary kidney tubular cells were collected using 0.05% trypsin (Wisent, Inc., Saint-Jean-Baptiste, QC), washed with PBS and suspended in binding buffer. The cells were double stained with Annexin V and propidium iodide (PI) using the FITC Annexin V Apoptosis Detection Kit (BD Biosciences, Mississauga, ON) following the manufacturer’s protocols and detected by flow cytometry using the FACSCalibur (BD Biosciences, San Diego, CA).

### Statistical Analysis

Data were collected as mean ± SEM. Statistical analysis on all quantitative data was performed using GraphPad Prism. A one way analysis of variance (ANOVA) was used to compare multiple treatment groups. P values < 0.05 were considered significantly different. Where necessary, a Tukey’s multiple comparison test was used to detect where the significant differences lie. A Log-rank (Mantel-Cox) Test was used to perform statistical analysis of animal survival data.
